# HIV-Related Stigma Among Healthcare Providers in Different Healthcare Settings: A Cross-Sectional Study in Kerman, Iran

**DOI:** 10.15171/ijhpm.2019.92

**Published:** 2019-10-21

**Authors:** Fatemeh Tavakoli, Mohammad Karamouzian, Ali Ahmad Rafiei-Rad, Abedin Iranpour, Mehrdad Farrokhnia, Mehdi Noroozi, Ali Sharifi, Brandon D.L. Marshall, Mostafa Shokoohi, Hamid Sharifi

**Affiliations:** ^1^HIV/STI Surveillance Research Center, and WHO Collaborating Center for HIV Surveillance, Institute for Futures Studies in Health, Kerman University of Medical Sciences, Kerman, Iran.; ^2^School of Population and Public Health, University of British Columbia, Vancouver, BC, Canada.; ^3^Department of Sociology, Allameh Tabatabai University, Tehran, Iran.; ^4^Neuroscience Research Center, Institute of Neuropharmacology, Kerman University of Medical Sciences, Kerman, Iran.; ^5^Department of Internal Medicine, Afzalipour School of Medicine, Kerman University of Medical Sciences, Kerman, Iran.; ^6^Substance Abuse and Dependence Research Center, University of Social Welfare and Rehabilitation Sciences, Tehran, Iran.; ^7^Department of Ophthalmology, Afzalipour School of Medicine, Kerman University of Medical Sciences, Kerman, Iran.; ^8^Department of Epidemiology, Brown University School of Public Health, Providence, RI, USA.; ^9^Department of Epidemiology & Biostatistics, The University of Western Ontario, London, ON, Canada.; ^10^Division of Social and Behavioural Health Sciences, Dalla Lana School of Public Health, University of Toronto, Toronto, ON, Canada.

**Keywords:** HIV, Stigma, Healthcare Providers, Kerman, Iran

## Abstract

**Background:** Stigmatizing attitudes among healthcare providers are an important barrier to accessing services among people living with HIV (PLHIV). This cross-sectional study aimed to assess the status and correlates of HIV-related stigma among healthcare providers in Kerman, Iran.

**Methods:** Using a validated and pilot-tested stigma scale questionnaire, we measured HIV-related stigma among 400 healthcare providers recruited from three teaching hospitals (n=363), private sectors (n=28), and the only voluntary counseling and testing (VCT) center (n=9) in Kerman city. Data were gathered using self-administered questionnaires at participants’ workplace during Fall 2016. To examine the correlates of stigmatizing attitudes, we constructed bivariable and multivariable linear regression models.

**Results:** The mean ± standard deviation (SD) of stigma score was 25.95 ± 7.20 out of the possible 50, with higher scores reflecting more stigmatizing attitudes. Paramedics, nurses’ aides, and housekeeping staff had the highest, and VCT personnel had the lowest average stigma scores, respectively. Multivariable regression analyses showed that prior experience of working with PLHIV (β=-2.48; *P*=.03), exposure to HIV-related educational courses (β=-2.03; *P*=.02), and <10 years of work experience (β=-2.70; *P*<.001) were associated with lower stigma scores.

**Conclusion:** Our findings highlight the need for health managers to provide training opportunities for healthcare providers, including programs that focus on improving HIV-related knowledge for healthcare providers. Enforcing policies that aim to reduce HIV-related stigma and discrimination among healthcare providers in Iran are urgently needed.

## Background

Worldwide, over 36 million are people living with HIV (PLHIV); ~60% of whom are linked to healthcare services.^1^ One of the critical barriers for PLHIV’s healthcare service utilization is HIV-related stigma (ie, a process of devaluation of people either living with or associated with HIV and AIDS within healthcare settings).^2^ Experiencing HIV-related stigma and discrimination among PLHIV is associated with a number of adverse health outcomes including feelings of anxiety, fear, frustration, depression, stress, shame, rejection, self-isolation as well as limited access to treatment and healthcare services.^3-7^

In the conservative context of the Middle East and North Africa, the adverse health outcomes of HIV-related stigma are significant and have led to low rates of HIV disclosure and case identifications.^8^ In Iran, for example, despite the expansion of care and treatment services for PLHIV in recent years, only about 30% of the estimated number of PLHIV in the country (~61 000) have been identified and connected to HIV care and treatment services.^9^ A failure that could be partly explained by the profound levels of HIV-related stigma experienced by PLHIV within their family, friends, workspace and the healthcare system that may drive them underground and leave them undetected.^10,11^

HIV-related stigma in healthcare settings is portrayed at individual- (eg, personal attitudes, beliefs, and behaviors), clinical- (eg, clinic characteristics, type, and location), and structural- (eg, institutional policies, support, and training) levels. Negative HIV-related attitudes and stigmatizing practices within healthcare providers have been associated with providers’ older age, lower levels of education, personal beliefs, and limited contact with PLHIV, as well as clinics’ rurality, and the unavailability of post-exposure prophylaxis. Moreover, HIV-related stigma has been reported to be lower within healthcare settings where institutional stigma reduction policies are in place and enforced.^12-15^

While the body of evidence on HIV-related stigma within healthcare settings in Iran is narrow, previous small-scale qualitative studies have reported frequent discriminatory attitudes toward PLHIV within healthcare settings in various forms of care refusal, sub-optimal care, excessive precautions, physical distancing, humiliation, and blaming.^10,16^ Higher levels of stigmatizing attitudes toward PLHIV in Iran have been associated with healthcare providers’ religious beliefs, fear of contracting HIV, and insufficient knowledge of transmission routes.^4,16^ While previous qualitative studies have been helpful in shedding light on the experiences of PLHIV’s interactions with healthcare providers, our understanding of HIV-related stigma within the Iranian healthcare system remains limited, given the restricted generalizability of previous qualitative studies and their focus on assessing HIV-related stigma from PLHIV’s perspectives.^10,16^ Given the significant role of healthcare providers in reaching PLHIV in Iran as well as supporting HIV national program in reaching the Joint United Nations Programme on HIV/AIDS 90-90-90 targets, the objective of this study is to quantify stigmatizing attitudes toward PLHIV from the perspective of healthcare providers to offer insight for future interventions aimed at addressing HIV-related stigma across healthcare settings. Quantitative assessment of HIV-related stigma could also help practitioners, policy-makers, and funding agencies in evaluating their HIV care and treatment programs and services.^15-17^

## Methods

### Participants and Sampling

A convenience sample of 400 healthcare providers in the city of Kerman located in the southeast of Iran was recruited for the study. Participants were eligible if they had at least one year of work experience and provided consent for participation in the study. Assuming that 42% of the healthcare providers had a fear of contracting HIV/AIDS,^4^ 95% power, a design effect of 1.5 (due to the clustered nature of the sample), and a precision level of 0.15, a sample size of 347 was calculated. Allowing for a non-response rate of 10% (347/(1–0.10)  =  385) and informed by expert opinions, the estimated sample size was adjusted to 400.

Sampling was carried out using a non-probability sampling approach between September to November 2016. Healthcare providers were recruited from 3 teaching hospitals of Afzalipoor, Shafa, and Shahid Bahonar in Kerman. Moreover, a sample of specialists and dentists from private practice, as well as the staff of the only HIV voluntary counseling and testing (VCT) center in Kerman, were included. To select individuals, we first took the number of people working in each sector and then chose them according to the proportion of people in each sector. For the private sector, we obtained the list of active specialists from the Islamic Republic of Iran Medical Council. We then chose the eligible specialists from the list. Participants completed a self-administered questionnaire delivered to them at their workplace.

### Questionnaire

We adopted the United States Agency for International Development Health Policy Project’s field-tested questionnaire developed by Nyblade et al.^17^ The items were presented in 4 scales: (1) Stigma toward PLHIV; (2) Worry scale of healthcare providers including 4 sub-scales: worry related to providing services to PLHIV, worry related to being avoided by family and co-workers, composite worry scale, and total worry scale; (3) Work environment policies; and (4) Attitudes related to pregnant women living with HIV. For analysis, each of the responses was categorized to agree (ie, agree and strongly agree), disagree (ie, disagree and strongly disagree), and no idea categories.^14^ We pilot-tested the questionnaire with 30 participants in the sampling framework across different specialties to ensure clarity, relevance, and accessibility. We also assessed content validity using the item content validity index. A panel of 20 experts reviewed the questionnaire for relevance and clarity using a Likert scale and items with content validity values of less than 0.78 were removed. Internal reliability of the questionnaire was assessed by measuring the Cronbach α coefficient and values >0.7 were considered acceptable.^18^

### Variables

#### Outcome Variable

The stigma scale included ten items which were scored using a 5-point Likert scale (1 = strongly disagree to 5 = strongly agree) with higher scores indicating greater levels of stigma. For analysis, these items were summed to create a continuous scale which could range from 0 to 50.^17^

#### Explanatory Variables

Explanatory variables included a range of socio-demographic variables such as gender, age, marital status, education, occupation, work experience, workplace setting, experience of working with PLHIV, and having passed HIV-related educational courses.

Other explanatory variables included worry scale of healthcare providers, work environment policies, and attitudes toward pregnant women living with HIV. The worry scale of healthcare providers had 4 sub-scales: (*a* ) Worry related to providing services to PLHIV, (*b* ) Worry related to being avoided by family and co-workers, (*c* ) Composite worry scale which consisted of the 2 previous scales, (*d* ) Total worry which consisted of the previous scales and items measuring worry about ‘being present at childbirth for a mother with HIV.’ All of the items were scored using a 5-point scale (1 = Not worried, 2 = A little bit worried, 3 = Worried, 4 = Very worried, 5 = Irrelevant). The first sub-scale of worry of the healthcare providers was worry related to providing services to PLHIV. This sub-scale consisted of 5 items. For analysis, these items were summed to create a continuous scale (Cronbach α = 0.881) which could range from 0 to 20. The second sub-scale was worry related to being avoided by family and co-workers which consisted of 2 items. For analyses, these items were summed to create a continuous scale (Cronbach α = 0.934) which could range from 0 to 8. The third sub-scale was the composite worry scale which consisted of 2 previous scales (Cronbach α = 0.823) and could range from 0 to 28. The last sub-scale was the total worry which consisted of the previous scales and items measuring concern about ‘being present at childbirth for a mother with HIV,’ which was scored in a 5-point scale (Cronbach = 0.857) and could range from 0 to 32. In all of these sub-scales, higher scores indicated greater levels of worry.

Another explanatory variable was work environment policies which consisted of 3 items. These items were scored using a 5-point Likert scale (1 = strongly agree, 2 = agree, 3 = No idea, 4 = disagree, 5 = strongly disagree). This scale could range from 0 to 15, so that higher scores indicated greater levels of worry about policies that were not protective (Cronbach α = 0.508).

The last explanatory variable was attitudes toward pregnant women living with HIV which consisted of 3 items. These items were scored using a 5-point Likert scale (1 = strongly agree, 2 = agree, 3 = No idea, 4 = disagree, 5 = strongly disagree). This scale could range from 0 to 15, so that higher scores indicated more stigmatizing attitudes (Cronbach α = 0.570).

### Data Analysis

Descriptive statistics were reported to estimate the stigma score. To assess the correlates of stigma, bivariable and multivariable linear regression models were used. Correlation analysis of the explanatory variables was conducted to identify collinear variables. Bivariable analysis was carried out to identify the correlates that were associated with stigma at *P* ≤ .25. Subsequently, all previously selected correlates were simultaneously entered into a full model which was reduced following a backward elimination approach using partial F-test. Data analysis was performed using svyset package in Stata software (StataCorp. 2015, Stata Statistical Software: Release 14.2 College Station, TX: StataCorp LP).^18,19^

## Results

The socio-demographic characteristics of the participants are presented in Table 1. The mean ± standard deviation )SD) age of the participants was 34.19 ± 8.92 years. Most participants were women (76.2%), married (77.1%), and nurses (40.2%). Moreover, most participants had a bachelor or associate degree (58.5%), had prior experience of providing care for PLHIV (63.0%), and had received HIV-related training (61.7%).

**Table 1:** Socio-demographic Characteristics to Estimate the HIV Stigma Among Health Providers in Kerman, Iran (n = 400)

**Variables**	**No. (%)**
Gender	
Men	95 (23.8)
Women	305 (76.2)
Age	
≤30	157 (41.0)
31-40	143 (37.3)
41-49	57 (14.9)
≥50	26 (6.8)
Marital status	
Married	307 (77.1)
Never married/single	91 (22.9)
Education	
Diploma or less	64 (16.0)
Bachelor/associate degree	234 (58.5)
MSc/MD/PhD	102 (25.5)
Occupation	
Medical specialist	26 (6.5)
Dentist	38 (9.5)
Nurse	161 (40.2)
Midwife	51 (12.7)
Laboratory worker	46 (11.5)
Paramedic, nurses’ aides, housekeeping staff	69 (17.4)
VCT personnel	9 (2.2)
Work experience	
<10	180 (51.0)
≥10	173 (49.0)
Workplace setting	
Educational hospitals	349 (87.3)
Private office/clinic	42 (10.5)
VCT clinic	9 (2.2)
Prior experience of working with PLHIV	
No	148 (37.0)
Yes	252 (63.0)
Having passed HIV-related educational courses	
No	149 (38.3)
Yes	240 (61.7)

Abbreviations: VCT, voluntary counselling and testing; PLHIV, people living with HIV.

Attitude toward PLHIV and worry related to providing services to PLHIV are presented in Tables 2 and 3. The mean ± SD score on the stigma scale was 25.95 ± 7.20 (range = 0–50). The findings bivariable and multivariable linear regression models are presented in Tables 4 and 5. Prior experience of working with PLHIV (β = -2.48, *P*  = .03), having passed educational courses related to HIV and other blood-borne diseases (β = -2.03, *P*  = .02), and work experience of less than 10 years were significant correlates of lower stigma scores (β = -2.70, *P* < .001).

**Table 2:** Attitudes Toward PLHIV Among Healthcare Providers in Kerman, Iran (n = 400)

**Level of Agreement**	**Disagree**	**No Idea**	**Agree**	**Missing**
Most PLHIV do not care if they infect others	99 (24.8)	140 (35.0)	150 (38.1)	9 (2.1)
PLHIV should feel ashamed of themselves	322 (80.5)	51 (12.8)	20 (5.1)	7 (1.6)
Most PLHIV have or have had many sexual partners	222 (55.6)	110 (27.5)	62 (15.5)	6 (1.4)
People get infected with HIV because they engage in irresponsible behaviors	196 (49.0)	106 (26.5)	92 (23.1)	6 (1.4)
HIV is a punishment for bad behavior	302 (75.5)	68 (17.0)	27 (6.8)	3 (0.7)
Women living with HIV should be allowed to have children if they wish	148 (37.1)	96 (24.0)	152 (38.0)	4 (0.9)
If I had a choice, I would prefer not to provide services to people who inject drugs	251 (62.8)	74 (18.5)	71 (17.8)	4 (0.9)
If I had a choice, I would prefer not to provide services to men who have sex with men	150 (37.6)	91 (22.8)	154 (38.5)	5 (1.1)
If I had a choice, I would prefer not to provide services to male sex workers	153 (38.3)	97 (24.3)	146 (36.5)	4 (0.9)
If I had a choice, I would prefer not to provide services to female sex workers	177 (44.3)	89 (22.3)	128 (32.0)	6 (1.4)

Abbreviation: PLHIV, people living with HIV.

**Table 3:** Worry Related to Providing Services to PLHIV and Being Avoided by Family and Co-workers (n = 400)

**Level of Worry When Conducting the Following Activities**	**Sub-scales**	**Not Worried and a Little Worried**	**Worried and Very Worried**	**Irrelevant**	**Missing**
Touching PLHIV’s clothes	Worry related to providing services to PLHIV	287 (71.8)	51 (12.8)	58 (14.5)	4 (0.9)
Dressing the wounds of PLHIV	Worry related to providing services to PLHIV	156 (39.3)	173 (43.0)	63 (15.8)	8 (1.9)
Drawing blood from PLHIV	Worry related to providing services to PLHIV	149 (37.3)	182 (45.5)	63 (15.8)	6 (1.4)
Measuring the temperature of PLHIV	Worry related to providing services to PLHIV	299 (74.8)	28 (7.0)	67 (16.8)	6 (1.4)
Providing health services for PLHIV (eg, dental fillings, surgery, and lavage)	Worry related to providing services to PLHIV	111 (27.8)	167 (41.8)	116 (29.0)	6 (1.4)
Friends and family avoiding you because you provide care for PLHIV	Worry related to being avoided by family and co-workers	214 (53.5)	28 (7.0)	151 (37.8)	7 (1.7)
Colleagues avoiding you because you provide care for PLHIV	Worry related to being avoided by family and co-workers	220 (55.1)	26 (6.5)	147 (36.8)	7 (1.6)

Abbreviation: PLHIV, people living with HIV.

**Table 4:** Bivariable Regression Results for Predicting Healthcare-Related HIV Stigma in Kerman, Iran (n = 375)^a^

Variable	**Stigma Score**		
	**Mean Score (95% CI)**	**Unadjusted Coefficient (95% CI)**	***P*** **Value**
Gender			
Men	25.46 (22.29, 28.63)	-	-
Women	26.10 (24.79, 27.41)	0.64 (-2.99, 4.27)	.65
Age			
≤30	25.27 (24.28, 26.25)	-	-
31-40	26.56 (24.36, 28.76)	1.29 (-1.78, 4.36)	.30
41-49	25.94 (21.95, 29.92)	0.66 (-3.48, 4.82)	.67
≥50	27.00 (24.99, 29.00)	1.72 (-0.41, 3.86)	.08
Marital status			
Married or divorce	25.98 (25.79, 26.18)	-	-
Never married/single	25.90 (24.51, 27.29)	-0.08 (-1.53, 1.36)	.87
Education			
Diploma or less	28.23 (25.42, 31.04)	-	-
Bachelor/associate degree	25.80 (24.74, 26.85)	-2.43 (-4.48, -0.37)	.03
MSc/MD/PhD	25.01 (22.45, 27.56)	-3.22 (-7.46, 1.02)	.10
Occupation			
Medical specialist	24.34 (20.92, 27.77)	-	-
Dentist	26.85 (26.85, 26.85)	2.50 (-0.91, 5.93)	.11
Nurse	25.92 (25.76, 26.09)	1.58 (-1.68, 4.84)	.25
Midwife	25.18 (23.10, 27.19)	0.83 (-2.58, 4.26)	0.53
Laboratory worker	26.30 (22.71, 29.90)	1.96 (-3.26, 7.19)	.35
Paramedic, nurses’ aides, housekeeping staff	28.11 (26.10, 30.11)	3.76 (-1.24, 8.78)	.10
VCT personnel	15.66 (12.76, 16.57)	-8.67 (-12.10, -5.25)	<.001
Work experience			
≤10	27.00 (24.45, 29.54)	-	-
>10	25.13 (23.75, 26.51)	-1.86 (-4.10, 0.37)	.08
Workplace setting			
Educational hospitals	26.16 (25.32, 27.00)	-	-
Private Clinic or office	26.57 (23.50, 28.00)	0.41 (-0.42, 1.24)	.24
VCT clinic	15.66 (12.76, 16.57)	-10.50 (-11.33, -9.66)	<.001
Prior experience of working with PLHIV			
No	27.46 (25.96, 28.96)	-	-
Yes	25.11 (23.79, 26.42)	-2.35 (-4.10, -0.60)	.02
Having passed HIV-related educational courses			
No	26.67 (25.89, 27.46)	-	-
Yes	25.31 (23.76, 26.87)	-1.36 (-2.58, -0.13)	.03
Worry related to providing services for PLHIV (n = 222)^b^	0.49 (0.29, 0.69)	<0.001	
Worry related to being avoided family and coworkers (n = 223)^b^	1.05 (0.08, 2.01)	0.03	
Composite worry scale (n = 140)^b^	0.49 (0.08, 0.91)	0.03	
Worry total (n = 50)^b^	0.27 (-0.22, 0.76)	0.17	
Environment policy (n = 367)	0.19 (-0.45, 0.84)	0.45	
Attitudes toward HIV-positive pregnant women (n = 154)^b^	-1.73 (-2.86, -0.61)	0.01	

Abbreviations: VCT, voluntary counselling and testing; PLHIV, people living with HIV. ^a^Some participants did not respond to all questions. ^b^These questions are not relevant for all participants and were not entered in the multivariable model.

**Table 5:** Multivariable Regression Results for Predicting Healthcare-Related HIV Stigma in Kerman, Iran (n = 375)^a^

**Variable**	**Adjusted Coefficient (95% CI)**	***P*** **Value**
Work experience		
≤10	-	-
>10	-2.70 (-4.13, -1.26)	<0.001
Prior experience of working with PLHIV		
No	-	-
Yes	-2.48 (-4.62, -0.33)	0.03
Having passed HIV-related educational courses		
No	-	-
Yes	-2.03 (-3.56, -0.49)	0.02

Abbreviation: PLHIV, people living with HIV. ^a^Some participants did not respond to all questions.

## Discussion

We conducted a study among healthcare providers in Iran, and found that HIV-related stigma was prevalent among them. We found that lower levels of stigma were associated with lower work experience, prior interactions with PLHIV, and having been exposed to HIV-related educational materials. Stigmatizing attitudes were more prevalent toward sexual practices rather than injecting behaviors which might be driven by the religious and conservative context of Iran as well as the relative socio-cultural acceptability of substance use in Kerman.^20^ Our findings are in line with previous qualitative studies in Iran^10,16,21^ and comparable with previous studies in other international settings such as Deep South,^14^ Nigeria,^22,23^ China,^24^ and Poland.^25^

In this study, we found no difference in most of the socio-demographic variables. These findings are contrary to other studies (eg, Deep South)^14^ which may be due to cultural differences across various settings. Stigmatizing attitudes however, were most prevalent among paramedics, nurses’ aides, and housekeeping staff which could be due to their lower levels of awareness about PLHIV. In the context of Iran, these groups often have lower levels of education compared to other healthcare professionals. Moreover, passing educational courses about HIV is not mandatory for these groups. Several studies have shown that higher levels of knowledge about transmission of HIV contribute to less fear from PLHIV and, therefore, reduced stigma. For example, less educated and less knowledgeable people about HIV have been shown to have more stigmatizing attitudes.^15,26,27^

Dentists and laboratory workers also had high levels of HIV-related stigma. This may be due to the nature of their jobs, which requires frequent contact with blood and other bodily fluids. The results of a study that examined the experiences of PLHIV in Iran also indicated that participants frequently faced stigmatizing attitudes when referring to a dental clinic.^5^ This finding is in contrast with the result of a study in Nigeria that reported doctors stigmatized PLHIV more than laboratory personnel and dentists in their setting which could be due to different work cultures and environments between Iran and Nigeria.^22^ Regardless, these findings highlight the importance of targeting these healthcare providers in HIV-related stigma reduction efforts and training programs.

Many healthcare workers expressed worry related to providing services to PLHIV. There were higher levels of worry related to actions such as wound management or contact with bodily fluids from PLHIV. This finding is in line with previous studies in Iran suggesting that healthcare providers’ stigmatizing attitudes were mainly driven by their fear of contracting HIV.^4,28^ Also, according to a study in Chicago, concerns were prevalent in comparison to other forms of HIV-related stigma.^6^ Interventions that reduce worry related to providing services to PLHIV and fear-based stigma through training on basic knowledge of HIV and universal precautions have been proven effective in reducing stigma and could be introduced in Iran to help improve the quality of care provided to PLHIV.^29^

Working experience with PLHIV, passing educational courses related to HIV and other blood-borne infections, and fewer years of work experience were associated with lower HIV-related stigma. Experience of working with PLHIV could reduce stigma and more frequent contact with PLHIV infected patients would lead to less stigmatizing attitudes.^7^ Passing relevant educational courses can also reduce stigma by increasing the knowledge of healthcare providers. This finding is in accordance with the results of a review that showed significant associations between reducing HIV-related stigma and knowledge about the transmission of HIV; These findings suggest the utility of programs providing opportunities for healthcare workers to learn about recognizing stigma and highlight the importance of confronting both fears of contagion and negative social judgments as ways to reduce HIV-related stigma among healthcare workers.^30^ Finally, those with less work experience had a lower stigma score. It seems those with less work experience have more HIV-related knowledge through their university training as well as professional development courses which could help improve their attitude and reduce stigma. Indeed, HIV-related trainings and course are relatively new in the Iranian educational context, and older healthcare workers may have not been exposed to them.^31^ Therefore, it is essential to ensure that all healthcare providers regardless of their work experience or seniority level, receive HIV-related training and learn about providing humane care for PLHIV. Overall, previous studies suggest the following steps for reduction of HIV-related stigma within healthcare settings: (*a* ) Using group discussion, games and role-playing to train popular opinion leaders, (*b* ) Interventions through peer groups, (*c* ) Group education in order to reduce fear of being infected, (*d* ) Interventions in healthcare setting through interactive modular training and discussion focusing on HIV-related stigma, infection control, medical ethics and contact with PLHIV, (*e* ) Workshops, training, and dissemination of policy guidelines and educational materials, such as posters on infection control.^32^ Implementing these interventions might help improve the quality of care for PLHIV in Iran.

We acknowledge the limitation of our study. Our study was conducted in a single large city which limits the generalizability of the findings to the rest of the country. However, considering the cultural context of Iran, we think our findings are informative for other provinces. Similar to studies in this area and on healthcare providers, our findings relied on self-reported responses and are subject to reporting and social desirability biases. Moreover, small sample sizes in most subgroups of providers limited our ability to make comparisons between different care provider groups. Nonetheless, this study represents one of the first studies in Iran and the Middle East that quantifies HIV-related stigma from the perspectives of healthcare providers and provides insight for future HIV-related stigma reduction interventions in the Iranian healthcare setting and beyond.

## Conclusion

We found HIV-related stigma among healthcare providers to be prevalent which calls for revisiting and revising the existing HIV-related stigma reduction policies and interventions in healthcare settings in Iran. Healthcare managers need to provide appropriate training and educational opportunities for healthcare providers to help reduce unnecessary worry when caring for PLHIV as well as HIV-associated stigmatizing attitudes. Ensuring the enforcement of policies aimed at reducing discrimination in various healthcare workplaces would be useful and help reduce HIV-related stigma among healthcare providers.

## Acknowledgements

The authors are grateful to Kerman University of Medical Sciences, Kerman, Iran for research support and all the staff who participated in the study. Also, MK is supported by the Vanier Canada Graduate Scholarship and the Pierre Elliott Trudeau Foundation Doctoral Scholarship.

## Ethical issues

The ethics committee of the Kerman University of Medical Sciences, Kerman, Iran reviewed and approved this study protocol (Reference number: IR.KMU.AH.REC.1395.40).

## Competing interests

Authors declare that they have no competing interests.

## Authors’ contributions

Design and conduct the survey: FT, MK, AI, MN, MS, HS. Data collection: FT. Data analysis: FT, AI, HS. Questionnaire validity and reliability assessment: AHRR, AI, MF, AS, HS. Supervision: HS, AS. Writing-original draft: FT, MK, MS, HS. Writing, review, and editing: FT, MK, AHRR, AI, MF, MN, AS, BDLM, MS, HS. Grant: AS, HS. All authors read the manuscript and approved the final version of the manuscript.

## Authors’ affiliations

^1^HIV/STI Surveillance Research Center, and WHO Collaborating Center for HIV Surveillance, Institute for Futures Studies in Health, Kerman University of Medical Sciences, Kerman, Iran. ^2^School of Population and Public Health, University of British Columbia, Vancouver, BC, Canada. ^3^Department of Sociology, Allameh Tabatabai University, Tehran, Iran. ^4^Neuroscience Research Center, Institute of Neuropharmacology, Kerman University of Medical Sciences, Kerman, Iran. ^5^Department of Internal Medicine, Afzalipour School of Medicine, Kerman University of Medical Sciences, Kerman, Iran. ^6^Substance Abuse and Dependence Research Center, University of Social Welfare and Rehabilitation Sciences, Tehran, Iran. ^7^Department of Ophthalmology, Afzalipour School of Medicine, Kerman University of Medical Sciences, Kerman, Iran. ^8^Department of Epidemiology, Brown University School of Public Health, Providence, RI, USA. ^9^Department of Epidemiology & Biostatistics, The University of Western Ontario, London, ON, Canada. ^10^Division of Social and Behavioural Health Sciences, Dalla Lana School of Public Health, University of Toronto, Toronto, ON, Canada.

## Key messages

**Implications for policy makers**

- Healthcare providers’ stigmatizing attitudes toward those affected through unsafe sexual practices are more prevalent than those affected via unsafe injection.
- Providing specific training opportunities about HIV among healthcare providers, paramedics, nurses’ aides, and housekeeping staff, in particular, may help reduce HIV-related stigma and discriminatory practices within healthcare settings in Iran.

**Implications for the public**

HIV-related stigma, in healthcare settings, in particular, is a significant barrier to HIV care and treatment efforts. We studied the stigmatizing attitudes of healthcare providers toward people living with HIV (PLHIV) in the southeast of Iran and found them to be prevalent. We have a moral and social responsibility to treat all patients - including PLHIV- with respect, dignity, and compassion to help reduce the burden of HIV within our communities.
